# The effects of meteorological factors and air pollutants on the incidence of tuberculosis in people living with HIV/AIDS in subtropical Guangxi, China

**DOI:** 10.1186/s12889-024-18475-0

**Published:** 2024-05-17

**Authors:** Fengyi Wang, Zongxiang Yuan, Shanfang Qin, Fengxiang Qin, Junhan Zhang, Chuye Mo, Yiwen Kang, Shihui Huang, Fang Qin, Junjun Jiang, Aimei Liu, Hao Liang, Li Ye

**Affiliations:** 1 Guangxi Key Laboratory of AIDS Prevention and Treatment, School of Public Health, Guangxi Medical University, Nanning, Guangxi China; 2 Joint Laboratory for Emerging Infectious Diseases in China (Guangxi)-ASEAN, Life Sciences Institute, Guangxi Medical University, Nanning, Guangxi China; 3 Chest Hospital of Guangxi Zhuang Autonomous Region, Liuzhou, China

**Keywords:** DLNM, Tuberculosis, HIV/AIDS, Meteorological factors, Air Pollutant

## Abstract

**Background:**

Previous studies have shown the association between tuberculosis (TB) and meteorological factors/air pollutants. However, little information is available for people living with HIV/AIDS (PLWHA), who are highly susceptible to TB.

**Method:**

Data regarding TB cases in PLWHA from 2014 to2020 were collected from the HIV antiviral therapy cohort in Guangxi, China. Meteorological and air pollutants data for the same period were obtained from the China Meteorological Science Data Sharing Service Network and Department of Ecology and Environment of Guangxi. A distribution lag non-linear model (DLNM) was used to evaluate the effects of meteorological factors and air pollutant exposure on the risk of TB in PLWHA.

**Results:**

A total of 2087 new or re-active TB cases were collected, which had a significant seasonal and periodic distribution. Compared with the median values, the maximum cumulative relative risk (RR) for TB in PLWHA was 0.663 (95% confidence interval [CI]: 0.507–0.866, lag 4 weeks) for a 5-unit increase in temperature, and 1.478 (95% CI: 1.116–1.957, lag 4 weeks) for a 2-unit increase in precipitation. However, neither wind speed nor PM_10_ had a significant cumulative lag effect. Extreme analysis demonstrated that the hot effect (RR = 0.638, 95%CI: 0.425–0.958, lag 4 weeks), the rainy effect (RR = 0.285, 95%CI: 0.135–0.599, lag 4 weeks), and the rainless effect (RR = 0.552, 95%CI: 0.322–0.947, lag 4 weeks) reduced the risk of TB. Furthermore, in the CD4(+) T cells < 200 cells/µL subgroup, temperature, precipitation, and PM_10_ had a significant hysteretic effect on TB incidence, while temperature and precipitation had a significant cumulative lag effect. However, these effects were not observed in the CD4(+) T cells ≥ 200 cells/µL subgroup.

**Conclusion:**

For PLWHA in subtropical Guangxi, temperature and precipitation had a significant cumulative effect on TB incidence among PLWHA, while air pollutants had little effect. Moreover, the influence of meteorological factors on the incidence of TB also depends on the immune status of PLWHA.

**Supplementary Information:**

The online version contains supplementary material available at 10.1186/s12889-024-18475-0.

## Background

Tuberculosis (TB) is a chronic airborne infectious disease that can cause damage to organs throughout the body, with pulmonary TB being the most common form [1]. There were an estimated 10.6 million cases of TB and approximately 1.6 million deaths attributed to TB in 2021 [2]. There are some few known risk factors that influence the development of TB disease, including diabetes, alcohol consumption, and drugs abuse [3]. Furthermore, meteorological conditions and air pollution may have a delayed and cumulative effect on the incidence of TB [4]. Previous studies have revealed that precipitation, temperature, PM_2.5_, PM_10_, SO_2_, O_3_, and CO may have a significant impact on TB prevalence [4–6]. PM_2.5_ and PM_10_ can carry *Mycobacterium tuberculosis* (*MTB*) into the lungs through inhalation, increasing the risk of *MTB* infection or worsening existing cases [6]. Gaseous pollutants, including SO_2_, O_3_, CO, and others, also indirectly contribute to the prevalence of TB by impairing lung health and weakening immune defences against *MTB* infection [7]. Therefore, exposure to high levels of these air pollutants would make it easier for a person to become infected with TB or would worsen existing TB. The incidence of TB also can be influenced by precipitation and temperature through modulation of the human immune response or change of human behavior [8, 9]. However, due to the differences in data quality, city-specific characteristics, and nations and populations, the effects of meteorological factors and air pollutants remain controversial [5, 7, 10–13].

People living with HIV/AIDS (PLWHA) are particularly vulnerable to developing TB owing to their weakened immune system [14]. TB accounts for approximately one-third of AIDS-related deaths globally, placing it as a leading cause of HIV-associated hospitalisation and mortality among PLWHA [15]. It is estimated that 187,000 PLWHA died from TB in 2021 [2]. As a result, HIV/TB coinfection has raised challenges for programme management and treatment around the world. At present, research on risk factors for TB in PLWHA mainly focuses on pathogens and host immunity [16, 17]. Limited attention has been paid to the cumulative effects of meteorological conditions and air pollution. Besides, the high susceptibility of PLWHA to TB can provide better research conditions to decipher the effects of meteorological factors and pollutants on TB incidence.

China is one of the top 30 countries regarding the burden of HIV/TB coinfection, and its annual TB incidence is the third highest in the world [18]. The Guangxi Zhuang Autonomous Region (Guangxi), a border province located in south-western China, has one of the highest burdens of HIV/TB coinfection in China [19, 20]. Guangxi is dominated by a subtropical monsoon climate with abundant heat and and precipitation, which provides favourable conditions for some bacterial and fungal pathogens, such as *MTB* [21] and *Talaromyces marneffei* [22]. Therefore, Guangxi is appropriate location to study of the correlation between meteorological factors and HIV/TB coinfection in a subtropical area.

To quantitatively assess the impact of meteorological conditions and air pollution on TB incidence, previous studies have commonly utilized a distributed lag non-linear model (DLNM) to evaluate the cumulative effects of exposure on the outcome incidence [5, 7, 10, 23]. However, limited research has been conducted on TB co-infected with HIV. A DLNM provides a framework that can be applied to describe the connections in time-series data that possibly have non-linear and delayed effects [24]. In this study, we used a DLNM to analyse the delayed and cumulative effects of meteorological factors and air pollutants on the incidence of TB among PLWHA in the subtropical areas of Guangxi. These findings will hopefully provide a reference for early warning and control of HIV/TB coinfection in this region.

## Materials and methods

### Research location

The Guangxi Zhuang Autonomous Region, a province including 14 cities, is located in south-western China. A total active temperature for the daily mean temperatures > 10 °C serves as the indicator to determine the demarcation line of a climatic zone, while the 6900℃ isotherm serves as the demarcation line between the south subtropic and middle subtropic [25, 26]. This study was conducted in nine cities: Nanning, Wuzhou, Chongzuo, Laibin, Yulin, Baise, Qinzhou, Fang Chenggang, and Guigang. Each city has a subtropical monsoon climate, with four distinct seasons, including a mild and wet winter, and a hot and rainy summer.

### Data sources

The data on the incidence of TB cases in PLWHA from2014to 2020 were collected from the HIV antiviral therapy cohort in subtropical areas of southern Guangxi, China. This study included 2087 new and re-active cases.

The meteorological data came from The China Meteorological Science Data Sharing Service Network (https://data.cma.cn/), including temperature (°C), wind speed (m/s), precipitation (mm), sunshine duration (h), and relative humidity (%). The atmospheric environment monitoring data came from the data center of the official website of the Department of Ecology and Environment of Guangxi Zhuang Autonomous Region (http://sthjt.gxzf.gov.cn/), including CO (mg/m^3^), O3 (µg/m^3^), NO_2_ (µg/m^3^), PM_2.5_ (µg/m^3^), and PM_10_ (µg/m^3^).

### Statistical analysis

Spearman correlation analysis was used to select the pertinent TB variables; the absolute correlation coefficient for relevant variables should be < 0.7 to minimise the collinearity issue [27]. Then characteristics of TB cases in PLWHA and the distribution of relevant meteorological factors and air pollutants are described as the median and interquartile range (IQR). A two-tailed *P*-value < 0.05 was considered to indicate a statistically significant difference.

The Kolmogorov-Smirnov test showed that the cases of TB among PLWHA approximately followed a Poisson distribution (data not shown). Therefore, based on a generalised additive model (GAM), the nonlinear relationship between the meteorological factors and air pollutants and the incidence of TB in PLWHA as well as the lag-response effect were analysed by using the cross-basis function [24]. A natural cubic spline (ns) function was used to control the other meteorological factors, air pollutants, and long-term trends [28]. The “week” variable (from 1 to 366 weeks) was used to regulate long-term trends and seasonal fluctuations. The meteorological factors and air pollutants were averaged on a weekly basis and then included in the model [29, 30]. Wind speed, temperature, precipitation, and PM_10_ were controlled by the ns function with three degrees of freedom [7, 10, 27].

Therefore, we employed a GAM based on “Quasipoisson” distribution to fit the overall effects of exposure, response, and lag effects [10, 31]. Using the natural cubic spline function as the basis function, air pollution and meteorological data were incorporated into the model. The DLNM model formula is as follows (taking temperature as an example):

$$ Y\_t{\mkern 1mu} \sim quasi{\mkern 1mu} Posisson(\mu \_t) $$

$$ \begin{gathered} Log(\mu \_t) = \alpha + \beta {\text{P}}\_(t,l) + {\text{ns}}\,({\text{time,df}}0) + {\text{ns}}\,({\text{windspeed}},\,{\text{df}}1) \\ + {\text{ns}}\,({\text{precipitation}},\,{\text{df}}2) + {\text{ns}}\,({\text{PM}}10,\,{\text{df}}4) \\ \end{gathered} $$

In this equation, Y_*t* is the number of cases in week “*t*”, α is the intercept, β is the coefficient of P_(*t*. *l)*, P_(*t*. *l)*is the temperature cross-basis matrix, “*l*” is lagging weeks, “ns” is the natural cubic spline function, and “df” is the degree of freedom. The “time” variable was used to control the long-term trend. The “df_0_” and lag weeks in the model were determined according to the Akaike information criterion (AIC). All mereological factors and air pollutants were controlled by “ns” function with three degrees of freedom [7, 32, 33]. Finally, a sensitivity analysis was carried out to test the stability of the results when changing the degrees of freedom of the time variable in the model.

## Results

### Characteristics of TB cases in PLWHA

The characteristics of all TB cases in PLWHA, meteorologic factors, and air pollution were shown in Table 1. A total of 2087 TB cases in PLWHA (males: 1699 [81.41%], females: 388 [18.59%]) were recorded in the Guangxi cohort of HIV antiviral therapy from 2014 to 2020. The median age was 48.55 years (IQR): 37.86–60.37 years); the median CD4(+) T-cell count was 68.00 cells/µL (IQR: 22.00-186.00 cells/µL); the median CD8(+) T-cell count was 546.00 cells/µL (IQR: 305.00-907.00 cells/µL); the median height was 163.96 cm (IQR: 160.00–168.00 cm); the median weight was 52.00 kg (IQR: 47.00–58.00 kg). The median and reference range of temperature, wind speed, precipitation, concentration of PM_10_ were 23.64 ℃ (IQR: 16.79–27.65 ℃), 1.85 m/s (IQR: 1.64–2.06 m/s), 3.30 mm (IQR: 1.24–7.11 mm), and 54.07 µg/m³ (IQR: 40.82–74.25 µg/m³), respectively.

**Table 1:** Characteristic of TB co-infected HIV cases, meteorological factors, and air pollutants in subtropical areas of southern Guangxi, 2014–2020

variables	Number (%)	Mean ± SD	Centiles									
			Min	1%	10%	25%	50%	75%	90%	99%	Max	IQR
**Demographic characteristic**												
All cases	2087(100)	5.70 ± 5.22	0.00	0.00	0.00	2.00	4.00	8.00	13.00	22.66	27.00	6.00
Male	1699(81.41)	5.20 ± 4.33	0.00	0.00	1.00	2.00	4.00	7.00	11.00	19.72	22.00	5.00
Female	388(18.59)	1.19 ± 1.34	0.00	0.00	0.00	0.00	1.00	2.00	3.00	5.00	7.00	2.00
CD4 < 200	1611(77.19)	4.40 ± 4.29	0.00	0.00	0.00	1.00	3.00	6.25	11.00	19.00	22.00	5.25
CD4 ≥ 200	476(22.81)	1.30 ± 1.44	0.00	0.00	0.00	0.00	1.00	2.00	3.00	6.00	8.00	2.00
Age (years)	-	49.10 ± 13.77	16.15	23.40	31.40	37.86	48.55	60.37	67.62	78.86	87.80	22.51
CD4 (+) T cells (cells/µL)	-	122.90 ± 138.54	0.00	0.00	10.00	22.00	68.00	186.00	306.20	579.24	1613.00	164.00
CD8 (+) T cells (cells/µL)	-	677.47 ± 576.10	0.00	0.00	163.80	305.00	546.00	907.00	1331.20	2530.84	10707.00	602.00
Height (cm)	-	163.96 ± 6.33	139.00	147.00	155.00	160.00	163.96	168.00	171.00	178.00	186.00	8.00
Weight (kg)	-	52.60 ± 8.45	29.00	35.00	42.00	47.00	52.00	58.00	63.00	76.12	96.00	11.00
**Meteorology factors in DLNM**												
Temperature (°C)	-	22.30 ± 5.83	7.61	9.98	13.52	16.79	23.64	27.65	28.66	29.66	30.12	10.86
Wind speed (m/s)	-	1.86 ± 0.30	1.16	1.23	1.47	1.64	1.85	2.06	2.24	2.60	2.73	0.42
Precipitation (mm)	-	4.71 ± 4.46	0.00	0.00	0.29	1.24	3.30	7.11	11.18	19.47	22.05	5.87
**Air pollutants in DLNM**												
PM_10_(µg/m3)	-	61.86 ± 29.67	16.71	22.77	33.10	40.82	54.07	74.25	99.33	168.56	206.80	33.43

Note: TB = tuberculosis; PM_10_ = particles with an aerodynamic diameter less than 10 μm; Min = minimum; Max = maximum; IQR = interquartile range; Mean ± SD = Mean plus or minus standard deviation

### The correlation between TB cases, meteorological factors, and air pollutants

**Fig. 1:**
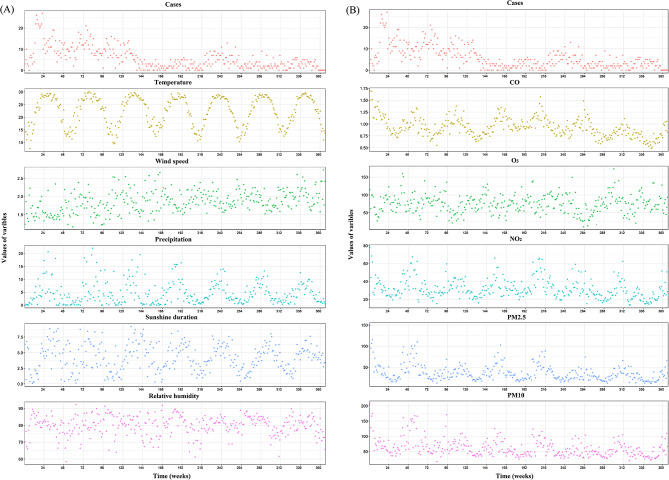
Time series of TB cases, meteorological factors (temperature, wind speed, precipitation, sunshine duration, and relative humidity) (**A**), and air pollutants (CO, O_3_, NO_2_, PM_2.5_, and PM_10_) (**B**) in subtropical Guangxi, China, from 2014 to 2020

In the time-series analysis, the TB cases, meteorological factors, and air pollutants had similar periodicity (Fig. 1). The variables related to the TB cases with correlation coefficients less than 0.7 with other factors were selected, and ultimately, 3 meteorological variables (i.e., temperature, wind speed, precipitation) and 1 air pollutant (i.e., PM10) were included in the DLNM for analysis.

To screen out the relevant variables for DLNM, Spearman correlation analysis was used to reveal a significant association between the cases and meteorological factors/air pollutants. CO, O_3_, NO_2_, PM_2.5_, sunshine duration, and relative humidity did not exhibit a significant correlation (*P* > 0.05) and thus were excluded from the model (Table S1). Considering that multicollinearity may affect the model’s stability [10], only the TB-related variables with a correlation coefficient to each variable of < 0.7 were included in the model. Thus, temperature (*r* = 0.234), wind speed (*r* = -0.292), precipitation (*r* = 0.157), and PM_10_ (*r* = 0.143) were incorporated in the DLNM(Table S1, *P* < 0.05). Among these variables, PM_10_ was positively correlated with TB and negatively correlated with the other variables (*P* < 0.01); wind speed was negatively correlated with TB and the other variables (*P* < 0.01); precipitation was positively correlated with TB and temperature (*P* < 0.01); and temperature was positively correlated with TB (*P* < 0.01).

### The effects of meteorological factors and air pollutant exposure on the risk of TB in PLWHA

**Fig. 2:**
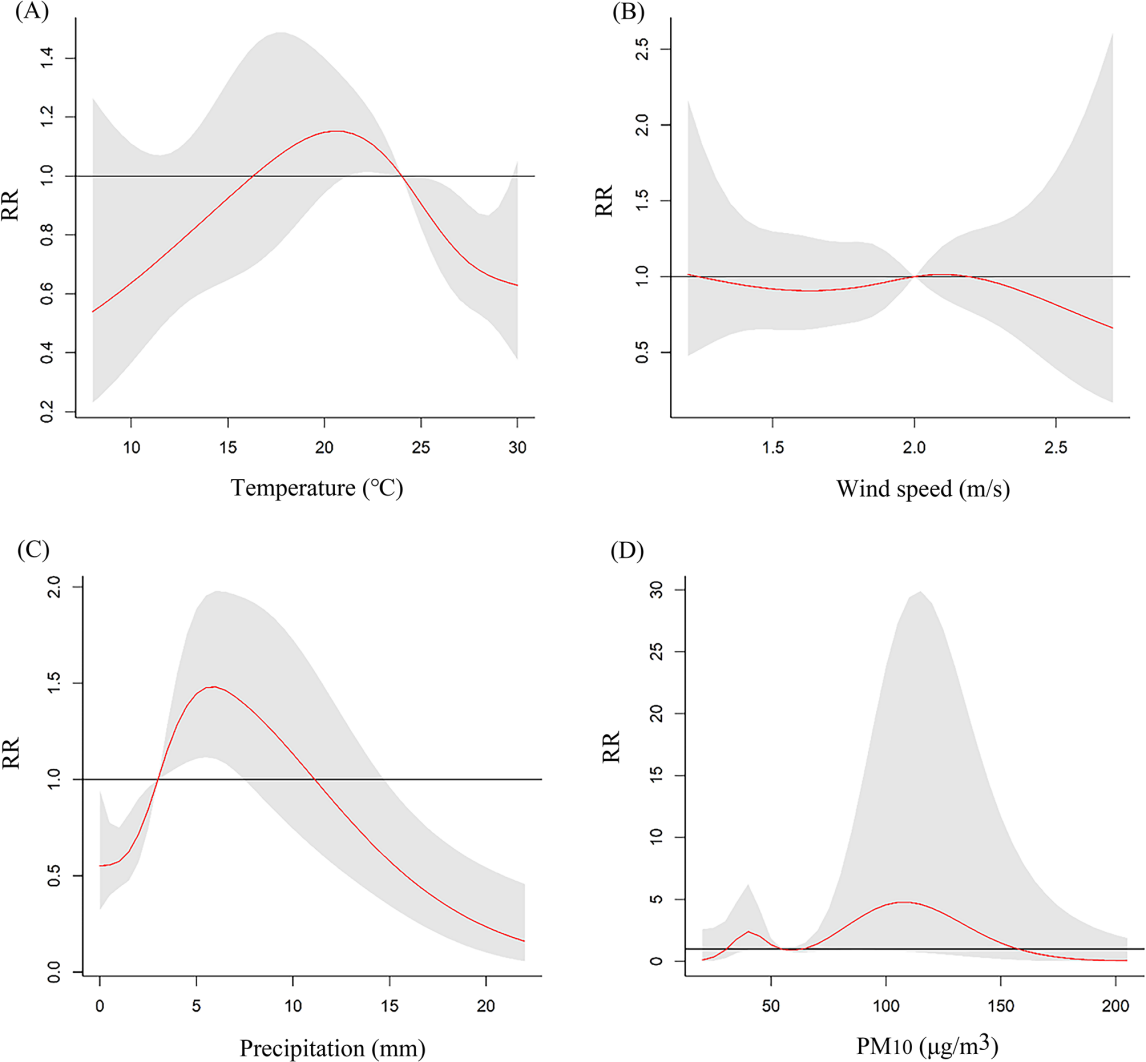
The overall exposure-response impact of temperature (**A**), wind speed (**B**), precipitation (**C**), and PM_10_ (**D**) on TB risk in PLWHA

**Fig. 3:**
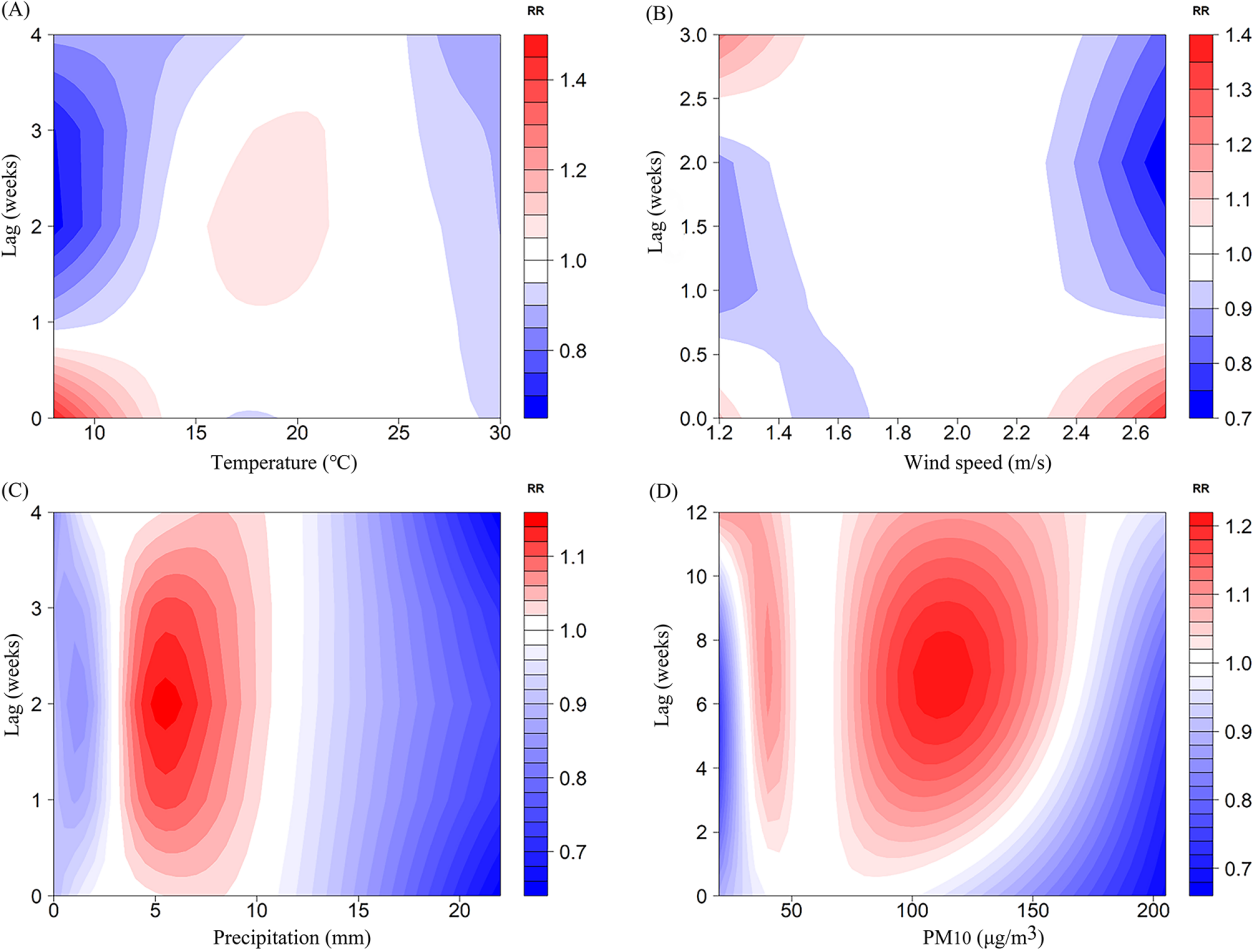
Contour plot of the effects of temperature (**A**), wind speed (**B**), precipitation (**C**), and PM_10_ (**D**)

**Fig. 4:**
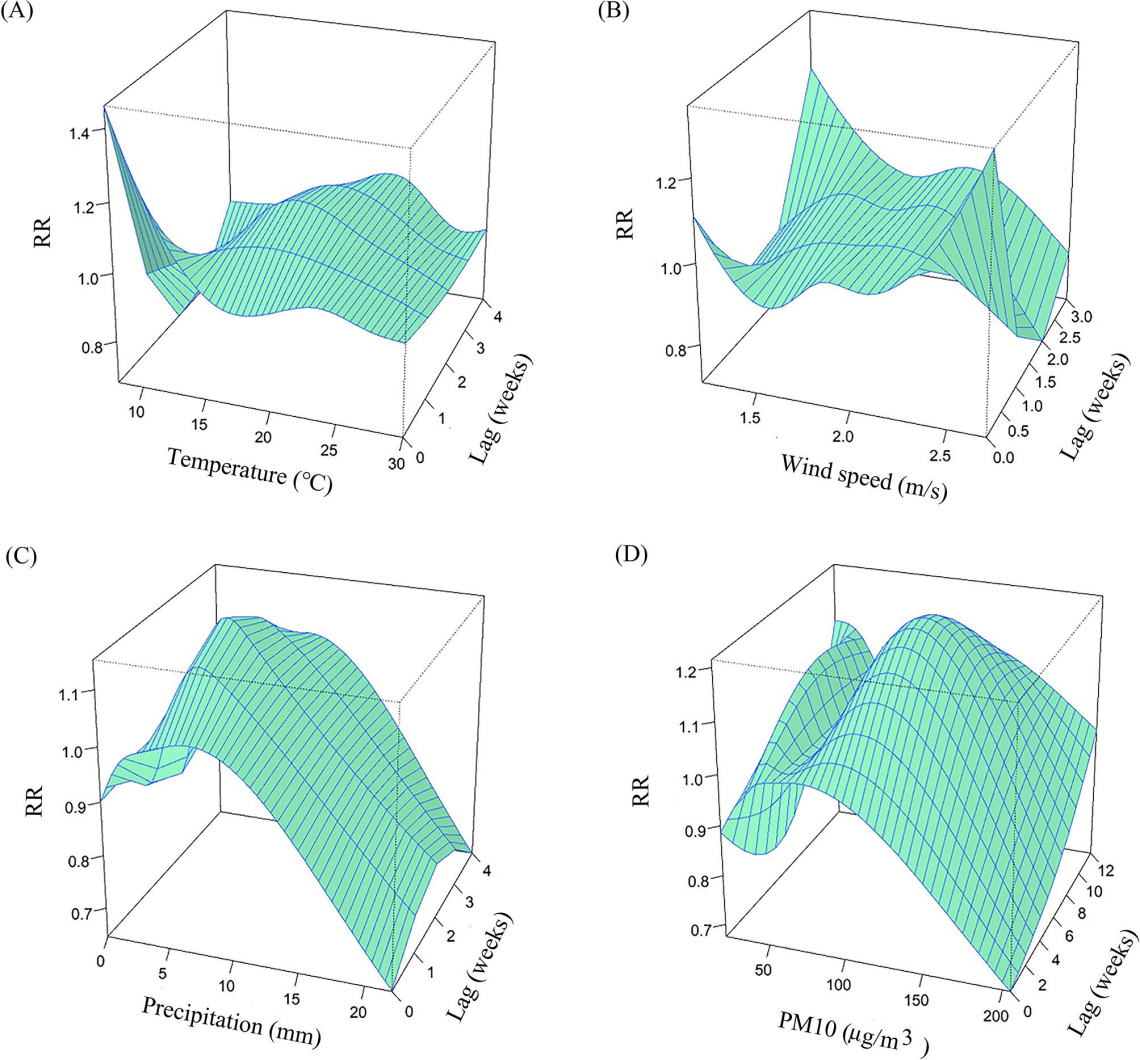
3D graph of the effects of temperature(A), wind speed (B), precipitation (C), and PM_10_ (D)

**Fig. 5:**
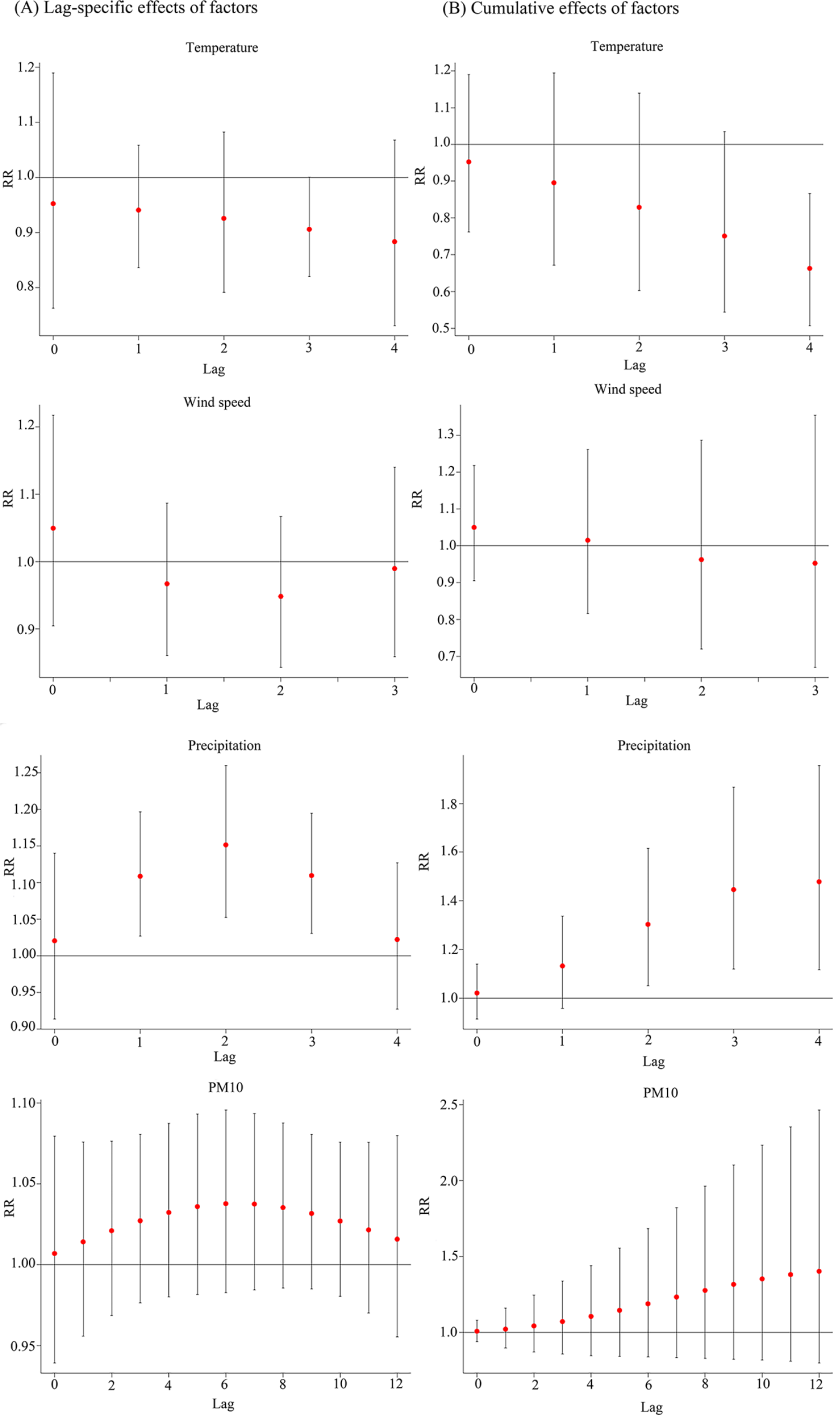
Lag-specific response effects for a unit increase in different factors (**A**); cumulative effects for lag-response incremental cumulative effects for a unit increase in different factors (**B**). In the model, a 5-unit increase for temperature, a 0.5-unit increase for wind speed, a 2-unit increase for precipitation, and a 15-unit increase for PM_10_ were used to calculate the relative risk of TB in PLWHA

**Fig. 6:**
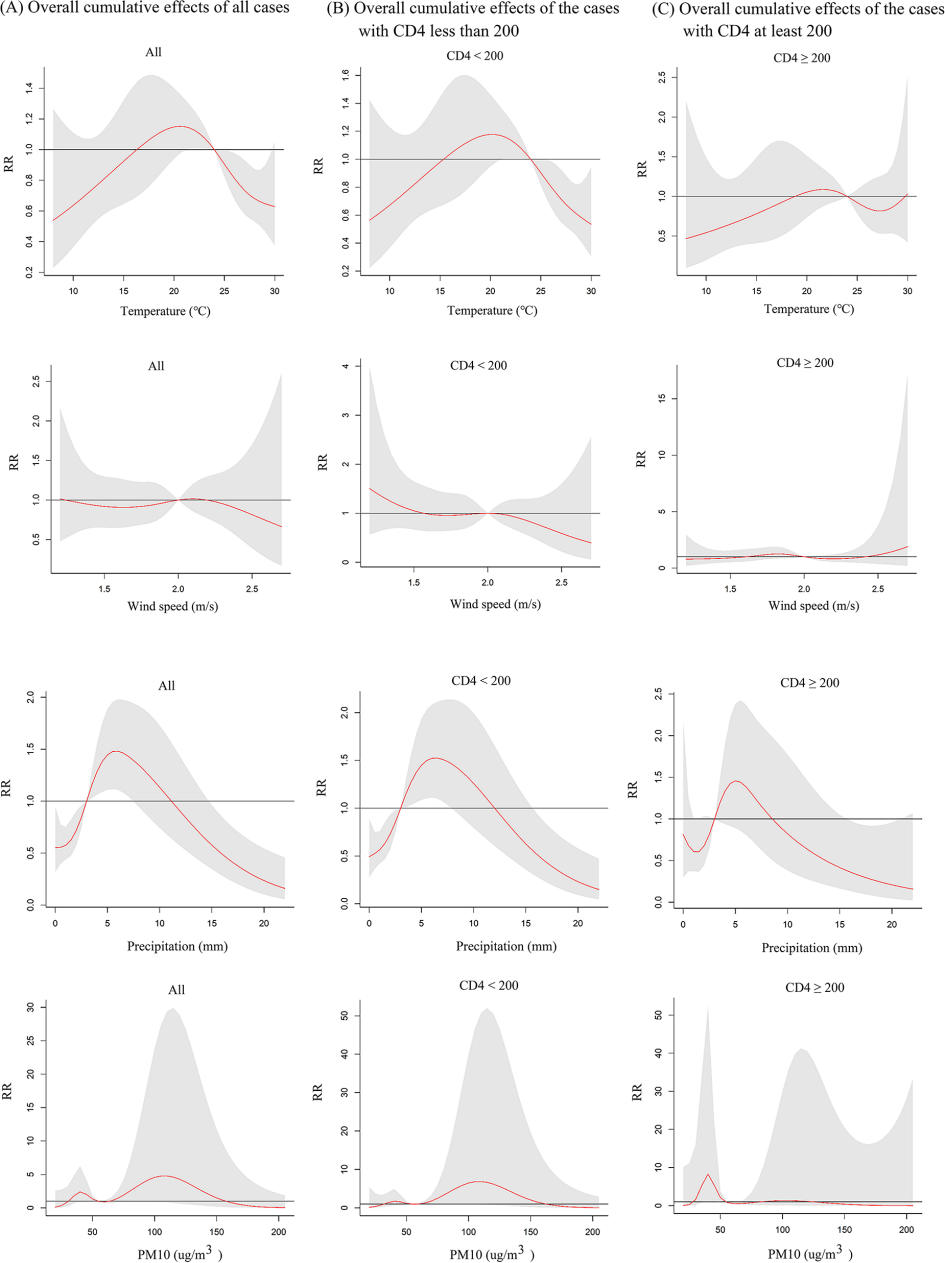
Summary of the overall cumulative association between TB incidence in people living with HIV/AIDS (PLWHA) and meteorological factors (temperature, wind speed, and precipitation) and air pollutants (PM_10_) in different subgroup, stratified by the CD4(+) T cell count

The lag times in the model were determined based on the AIC: a lag of 4 weeks for both temperature and precipitation, a lag of 3 weeks for wind speed, and a lag of 12 weeks for PM_10_. The overall exposure-response effects of meteorological factors and air pollutant exposure on TB risk in PLWHA were shown in Fig. 2. As temperature and precipitation increased, the relative risk (RR) showed a unimodal distribution, meaning that the risk of TB increased as both temperature and precipitation increased, and then decreased after they reached the peak. The RR distribution of PM_10_ was bimodal, with two peak points. The associations between meteorological factors and the lag time (in weeks) with the risk of TB in PLWHA were shown from two and three-dimensional perspectives in Figs. 3 and 4, respectively. To further explain the relationship between TB and the unit increase in the factors, compared with the medians, the cumulative RR of TB in PLWHA was calculated with the model (Table 2; Fig. 5). With a 5-unit increase in temperature, the cumulative RR was 0.663 (95% CI: 0.507–0.866, lag 4 weeks); with a 2-unit increase in precipitation, the RR of lag-response effect was 1.152 (95% CI: 1.052–1.260, lag 3 weeks), and the cumulative RR was 1.478 (95% CI: 1.116–1.957, lag 4 weeks). However, PM_10_ and wind speed had no significant effect on TB (*P* > 0.05). Stratified analysis was performed according to the CD4(+) T cells count [34–36] (shown in Tables 3 and 4; Fig. 6). In the CD4(+) T cells < 200 cells/µL subgroup, temperature (RR = 0.886, 95%CI: 0.794–0.987, lag 3 weeks), precipitation (RR = 1.167, 95%CI:1.058–1.287, lag 2 weeks), and PM_10_ (RR = 1.061, 95%CI:1.002–1.124, lag 7 weeks) had significant lag-response effects. Moreover, temperature (RR = 0.612, 95%CI: 0.457–0.819, lag 4 weeks) and precipitation (RR = 1.498, 95%CI: 1.105–2.032, lag 4 weeks) had the significant cumulative lag-response effects. In the CD4(+) T cells ≥ 200 cells/µL subgroup, there was no significant difference in either the lag-response effect or the cumulative effect.

**Table 2:** Distribution lag non-linear model results of TB cases with different meteorological factors and pollutant (Lag-response; Lag-response of incremental cumulative effects)

Lag (weeks)	Temperature				Wind speed					Precipitation					PM_10_				
	Lag-response^a^		Lag-response cumulative effects^a^		Lag-response^b^		Lag-response cumulative effects^b^			Lag-response^c^		Lag-response cumulative effects^c^			Lag-response^d^		Lag-response cumulative effects^d^		
**0**	0.952(0.762, 1.190)		0.952(0.762, 1.190)		1.050(0.905, 1.218)		1.050(0.905, 1.218)			1.020(0.913, 1.140)		1.020(0.913, 1.140)			1.007(0.939, 1.080)		1.007(0.939, 1.080)		
**1**	0.940(0.836, 1.058)		0.895(0.671, 1.195)		0.967(0.860, 1.087)		1.015(0.816, 1.262)			1.109(1.027, 1.197)*		1.131(0.958, 1.336)			1.014(0.956, 1.076)		1.021(0.898, 1.161)		
**2**	0.925(0.791, 1.083)		0.829(0.603, 1.140)		0.948(0.843, 1.067)		0.962(0.720, 1.287)			1.152(1.052, 1.260)*		1.303(1.051, 1.616)*			1.021(0.968, 1.076)		1.042(0.872, 1.246)		
**3**	0.906(0.820, 1.000)		0.750(0.544, 1.035)		0.990(0.859, 1.140)		0.953(0.670, 1.355)			1.110(1.031, 1.195)*		1.446(1.120, 1.867)*			1.027(0.976, 1.081)		1.071(0.857, 1.338)		
**4**	0.883(0.730, 1.068)		0.663(0.507, 0.866)*							1.022(0.927, 1.127)		1.478(1.116, 1.957)*			1.032(0.980, 1.087)		1.105(0.848, 1.440)		
**5**															1.036(0.982, 1.093)		1.145(0.843, 1.556)		
**6**															1.038(0.983, 1.096)		1.188(0.838, 1.684)		
**7**															1.037(0.984, 1.094)		1.233(0.834, 1.822)		
**8**															1.035(0.985, 1.088)		1.276(0.829, 1.964)		
**9**															1.032(0.985, 1.081)		1.316(0.824, 2.103)		
**10**															1.027(0.980, 1.076)		1.352(0.818, 2.234)		
**11**															1.021(0.970, 1.076)		1.381(0.810, 2.354)		
**12**															1.016(0.955, 1.080)		1.403(0.797, 2.467)		

Note: Relative risk (RR) with 95% confidence interval (CI) was used to describe the effect of the meteorological factors and pollutant factors in different subgroups. *P < 0.05

a The model is based on a maximum lag of 4 weeks, which was adjusted for long-term trend, wind speed, precipitation, and PM_10_. The Lag-response is for a 5-unit increase of temperature in the model

b The model is based on a maximum lag of 3 weeks, which was adjusted for long-term trend, mean temperature, precipitation, and PM_10_. The Lag-response is for a 0.5-unit increase of wind speed in the model

c The model is based on a maximum lag of 4 weeks, which was adjusted for long-term trend, mean temperature, wind speed, and PM_10_. The Lag-response is for a 2-unit increase of wind precipitation in the model

d The model is based on a maximum lag of 12 weeks, which was adjusted for long-term trend, mean temperature, wind speed, and precipitation. The Lag-response is for a 15-unit increase of wind PM_10_ in the model

**Table 3:** The analysis of Lag-response effects in different subgroup

Lag(weeks)	Temperature (℃)		Wind speed (m/s)		Precipitation (mm)		PM_10_ (µg/m3)		
	CD4 < 200	CD4 ≥ 200	CD4 < 200	CD4 ≥ 200	CD4 < 200	CD4 ≥ 200	CD4 < 200	CD4 ≥ 200	
**0**	0.950(0.746, 1.211)	0.959(0.642, 1.434)	1.004(0.851, 1.183)	1.061(0.832, 1.354)	0.996(0.882, 1.124)	1.094(0.896, 1.335)	1.014(0.939, 1.094)	0.985(0.860, 1.129)	
**1**	0.919(0.808, 1.046)	1.011(0.817, 1.251)	0.961(0.846, 1.093)	0.878(0.722, 1.067)	1.107(1.019, 1.203)*	1.116(0.970, 1.284)	1.024(0.960, 1.093)	0.981(0.874, 1.100)	
**2**	0.896(0.755, 1.064)	1.025(0.775, 1.357)	0.938(0.824, 1.067)	0.876(0.718, 1.068)	1.167(1.058, 1.287)*	1.112(0.943, 1.311)	1.034(0.976, 1.096)	0.976(0.882, 1.080)	
**3**	0.886(0.794, 0.987)*	0.975(0.817, 1.163)	0.931(0.793, 1.092)	1.053(0.838, 1.324)	1.127(1.040, 1.222)*	1.065(0.930, 1.220)	1.044(0.987, 1.103)	0.972(0.882, 1.071)	
**4**	0.882(0.717, 1.084)	0.892(0.632, 1.260)			1.033(0.929, 1.149)	0.997(0.835, 1.190)	1.051(0.993, 1.113)	0.968(0.876, 1.069)	
**5**							1.057(0.996, 1.122)	0.964(0.870, 1.069)	
**6**							1.061(0.999, 1.126)	0.961(0.865, 1.066)	
**7**							1.061(1.002, 1.124)*	0.958(0.866, 1.059)	
**8**							1.059(1.003, 1.118)*	0.955(0.869, 1.050)	
**9**							1.055(1.003, 1.110)*	0.953(0.872, 1.041)	
**10**							1.050(0.997, 1.105)	0.950(0.870, 1.039)	
**11**							1.043(0.986, 1.104)	0.948(0.859, 1.047)	
**12**							1.036(0.969, 1.108)	0.946(0.841, 1.065)	

Note: Relative risk (RR) with 95% confidence interval (CI) was used to describe the effect of the meteorological factors and pollutant factors in different subgroups. *P < 0.05

The temperature model is based on a maximum lag of 4 weeks, which was adjusted for long-term trend, wind speed, precipitation, and PM_10_. The Lag-response is for a 5-unit increase of temperature in the model

The wind speed model is based on a maximum lag of 3 weeks, which was adjusted for long-term trend, temperature, precipitation, and PM_10_. The Lag-response is for a 0.5-unit increase of wind speed in the model

The precipitation model is based on a maximum lag of 4 weeks, which was adjusted for long-term trend, temperature, wind speed, and PM_10_. The Lag-response is for a 2-unit increase of wind precipitation in the model

The PM_10_ model is based on a maximum lag of 12 weeks, which was adjusted for long-term trend, temperature, wind speed, and precipitation. The Lag-response is for a 15-unit increase of wind PM_10_ in the model

**Table 4:** The analysis of Lag-response cumulative effects in different subgroup

Lag(weeks)	Temperature (℃)		Wind speed (m/s)		Precipitation (mm)		PM_10_ (µg/m3)	
	CD4 < 200	CD4 ≥ 200	CD4 < 200	CD4 ≥ 200	CD4 < 200	CD4 ≥ 200	CD4 < 200	CD4 ≥ 200
**0**	0.950(0.746, 1.211)	0.959(0.642, 1.434)	1.004(0.851, 1.183)	1.061(0.832, 1.354)	0.996(0.882, 1.124)	1.094(0.896, 1.335)	1.014(0.939, 1.094)	0.985(0.860, 1.129)
**1**	0.874(0.639, 1.195)	0.970(0.576, 1.633)	0.965(0.758, 1.228)	0.931(0.660, 1.313)	1.102(0.919, 1.322)	1.220(0.902, 1.651)	1.038(0.902, 1.194)	0.966(0.753, 1.240)
**2**	0.783(0.554, 1.108)	0.994(0.559, 1.768)	0.905(0.657, 1.246)	0.815(0.516, 1.288)	1.287(1.018, 1.625)*	1.356(0.914, 2.012)	1.073(0.884, 1.304)	0.943(0.668, 1.332)
**3**	0.694(0.489, 0.985)*	0.969(0.543, 1.729)	0.842(0.570, 1.243)	0.859(0.503, 1.465)	1.450(1.099, 1.914)*	1.445(0.902, 2.315)	1.120(0.878, 1.429)	0.916(0.597, 1.408)
**4**	0.612(0.457, 0.819)*	0.865(0.534, 1.400)			1.498(1.105, 2.032)*	1.440(0.856, 2.421)	1.178(0.881, 1.574)	0.887(0.533, 1.475)
**5**							1.245(0.890, 1.743)	0.855(0.475, 1.538)
**6**							1.321(0.900, 1.937)	0.821(0.421, 1.601)
**7**							1.401(0.912, 2.153)	0.787(0.373, 1.660)
**8**							1.484(0.924, 2.384)	0.751(0.329, 1.712)
**9**							1.566(0.936, 2.620)	0.715(0.292, 1.752)
**10**							1.644(0.947, 2.854)	0.680(0.260, 1.777)
**11**							1.715(0.955, 3.082)	0.645(0.232, 1.791)
**12**							1.778(0.957, 3.304)	0.610(0.207, 1.801)

Note: Relative risk (RR) with 95% confidence interval (CI) was used to describe the effect of the meteorological factors and pollutant factors in different subgroups. *P < 0.05

The temperature model is based on a maximum lag of 4 weeks, which was adjusted for long-term trend, wind speed, precipitation, and PM_10_. The Lag-response cumulative effect is for a 5-unit incremental cumulative effect of temperature in the model

The wind speed model is based on a maximum lag of 3 weeks, which was adjusted for long-term trend, temperature, precipitation, and PM_10_. The Lag-response cumulative effect is for a 0.5-unit incremental cumulative effect of wind speed in the model

The precipitation model is based on a maximum lag of 4 weeks, which was adjusted for long-term trend, temperature, wind speed, and PM_10_. The Lag-response cumulative effect is for a 2-unit incremental cumulative effect of wind precipitation in the model

The PM_10_ model is based on a maximum lag of 12 weeks, which was adjusted for long-term trend, temperature, wind speed, and precipitation. The Lag-response cumulative effect is for a 15-unit incremental cumulative effect of wind PM_10_ in the model

### Extreme effect analysis of meteorological factors and air pollutants

The 99th and 1st percentiles were assigned as the extreme effect values for the meteorological factors and air pollutants; the extreme effect analysis results were presented in Table 5. The hot effect (99th vs. median, cumulative RR = 0.638, 95%CI: 0.425–0.958, lag 4 weeks), the rainy effect (99th vs. median, cumulative RR = 0.285, 95%CI: 0.135–0.599, lag 4 weeks), and rainless effect (1st vs. median, cumulative RR = 0.552, 95%CI: 0.322–0.947, lag 4 weeks) significantly reduced the risk of TB in PLWHA, but the extreme effects of the cold effect, wind speed, and PM_10_ were not significant (Table 5).

**Table 5:** Extreme effect analysis of different factors from 2014 to 2020

Lag (weeks)	Temperature		Wind speed		precipitation			PM_10_				
	Hot effect	Cold effect	Windy effect	Windless effect	Rainy effect		Rainless effect		High-PM10 effect		Low-PM10 effect	
**0**	0.948(0.705, 1.275)	1.273(0.874, 1.855)	1.263(0.812, 1.965)	1.112(0.795, 1.555)	0.741(0.565, 0.973)*		0.901(0.719, 1.129)		0.789(0.580, 1.072)		0.915(0.717, 1.168)	
**1**	0.877(0.596, 1.289)	1.201(0.734, 1.965)	1.097(0.580, 2.075)	0.949(0.577, 1.561)	0.586(0.391, 0.878)*		0.820(0.589, 1.140)		0.649(0.370, 1.138)		0.820(0.531, 1.267)	
**2**	0.795(0.520, 1.216)	0.939(0.542, 1.626)	0.842(0.359, 1.979)	0.834(0.431, 1.614)	0.478(0.286, 0.798)*		0.742(0.494, 1.117)		0.556(0.256, 1.206)		0.721(0.402, 1.293)	
**3**	0.714(0.461, 1.107)	0.732(0.412, 1.302)	0.736(0.261, 2.075)	1.015(0.475, 2.167)	0.380(0.203, 0.710)*		0.654(0.406, 1.053)		0.495(0.190, 1.292)		0.624(0.307, 1.269)	
**4**	0.638(0.425, 0.958)*	0.637(0.366, 1.110)			0.285(0.135, 0.599)*		0.552(0.322, 0.947)*		0.457(0.148, 1.411)		0.535(0.233, 1.226)	
**5**									0.434(0.119, 1.583)		0.458(0.176, 1.194)	
**6**									0.424(0.098, 1.826)		0.396(0.132, 1.192)	
**7**									0.421(0.082, 2.15)		0.350(0.100, 1.229)	
**8**									0.424(0.070, 2.562)		0.319(0.077, 1.315)	
**9**									0.430(0.060, 3.064)		0.302(0.062, 1.462)	
**10**									0.439(0.053, 3.667)		0.299(0.053, 1.697)	
**11**									0.449(0.046, 4.400)		0.312(0.047, 2.078)	
**12**									0.460(0.040, 5.336)		0.342(0.043, 2.715)	

Note: Relative risk (RR) with 95% confidence interval (CI) was used to describe the effect of the meteorological factors and pollutant factors on TB case occurrence in different subgroups. 99th percentiles and 1th percentiles were considered as the threshold of extreme effect. *P < 0.05

The temperature model is based on a maximum lag of 4 weeks, which was adjusted for long-term trend, wind speed, precipitation, and PM_10_.

The wind speed model is based on a maximum lag of 3 weeks, which was adjusted for long-term trend, mean temperature, precipitation, and PM_10_.

The precipitation model is based on a maximum lag of 4 weeks, which was adjusted for long-term trend, mean temperature, wind speed, and PM_10_.

The PM_10_ model is based on a maximum lag of 12 weeks, which was adjusted for long-term trend, mean temperature, wind speed, and precipitation

### Sensitivity analysis

Sensitivity analysis demonstrated that varying the degrees of freedom for a long-term trend of different factors in the DLNM did not lead to significant changes in the results, indicating that the findings are robust (Tables S2-S6).

## Discussion

HIV/TB co-infection remains a major public health challenge in China and throughout the world. With the development of bioinformatics methods and mathematical models, an increasing amount of research has been devoted to quantifying the impact of meteorological conditions on TB infection [5], but no relevant studies have been conducted in PLWHA. We applied a DLNM for the first time to investigate the association between environmental factors and TB incidence in PLWHA, who are susceptible to *MTB* infection. The findings suggest that meteorological conditions (including temperature and precipitation) as well as air pollutant (including PM_10_) may have a significant lag or cumulative impact on the TB incidence in PLWHA.

The results indicated that an increase in temperature may reduce the risk of TB in PLWHA, which is consistent with some previous studies [37–39]. A study conducted in Mexico indicated that TB has seasonality, with the highest incidence occurring during spring and summer seasons [40]. Rao et al. suggested that a 10 ℃ increase in temperature is associated with a 9% decrease in TB morbidity [41]. Therefore, temperature is considered a crucial influential factor in the context of TB. The biological mechanisms underlying the association between temperature and TB are complex. Increased temperatures have the potential to induce alterations in human behaviours [9]. Individuals tend to stay indoors within air-conditioned environment in high-temperature weather, thereby reducing the risk of close contact with TB patients [39]. Moreover, the host immune system may also be affected by lower temperatures [38]. Vitamin D deficiency is commonly observed during winter, which can potentially weaken immune function [42]. Therefore, elevated temperatures confer partical protection against TB, and it is interesting and noteworthy that meteorological factors may affect the host’s immune status, potentially establishing a correlation with TB. However, in our study, the cold effect was not statistically significant, possibly attributed to the relatively weak impact of cold weather in the subtropical region of Guangxi due to its warm climate, resulting in statistical insignificance.

The results also suggest that increased precipitation may increase the risk of TB in PLWHA, whereas rainy or rainless has the opposite effect. Increased precipitation could broaden the habitat range of *MTB* [43], and moist air facilitates the survival and reproduction of *MTB* [44]. Additionally, the rainless effect may exert a protective effect by inducing aridity in the environment, while inclement weather diminishes outdoor activities and consequently reduces the risk of transmission through direct interpersonal contact [45].

In the present study, there was no significant association between wind speed, PM_10_, and TB incidence in PLWHA. A few previous studies have indicated that increased wind speed could accelerate the spread of *MTB* [46] and change the distribution of air pollutants [47]. However, another studies showed that the wind reduced the TB risk because it dilutes the concentration of bacteria and air pollutants [48]. A study indicated that PM_10_ had an indirect impact on the incidence of TB, displaying a positive correlation with TB occurrence [7]. PM_10_ exposure causes the senescence of respiratory epithelial cells, reduces the expression of HBD-2 and HBD-3, and promotes the development of TB [49]. In other studies, there was no association between increased PM10 and TB incidence [11, 13], which consistent with our research. For one thing, the industrial development in Guangxi remains inadequate; thus, the air quality is commendable. For another thing, the association between PM_10_ and TB incidence depends on the PM_10_ concentration. Wang et al. [7] revealed a significant association between the two, but the median PM_10_ concentration in that study (139.00 µg/m³) was 2.57 times higher than the present study (54.07 µg/m³). A study conducted in South Korea [13] reported a similar concentration (63.50 µg/m³) as the present study, and those authors also did not establish a significant correlation between PM_10_ and TB incidence.

The factors of relative humidity, sunshine duration, O3 etc. were excluded from the model analysis in our study due to their lack of association with TB incidence in PLWHA. Some relevant literatures have reported significant delayed cumulative effects of relative humidity [38], sunshine duration [50], and O3 [51] on the incidence of TB. However, other studies have found that these meteorological factors and air pollutants do not have a significant impact on the incidence of TB [5, 33, 52]. Therefore, variations in data quality, region specific characteristics, and populations contribute to differences in the effects observed [5, 7].

Climate change impacts TB through various pathways, alterations in climatic factors such as temperature and precipitation influence the host immune responses by modifying the distribution of vitamin D, Ultraviolet (UV) radiation exposure, and other risk factors [8]. Interestingly, we found the influence of meteorological factors on the TB incidence related with the immune status of PLWHA. A CD4(+) T cell count below 200 cells/µL indicates a diagnosis of AIDS after HIV infection [53], signifying severe impairment of the immune system [54]. In the present study, we found that patients with compromised immune system were more sensitive to the effects on climate change and air pollutants. Alterations in meteorological factors can impact the susceptibility to TB by modulating the host immune response. An increase in precipitation results in a humid surrounding environment and a reduction in UV radiation. UV radiation serves as the primary source of Vitamin D, which exhibits immunostimulatory and immunosuppressive effects associated with anti-mycobacterial responses in humans [55]. The deficiency of Vitamin D is correlated with an elevated susceptibility to TB [56]. Vitamin D deficiency is more prevalent among PLWHA, because it is correlated with compromised immune responses in PLWHA [57]. Therefore, PLWHA is more sensitive to climate fluctuations in TB incidence than the general population, particularly among those with severe immune dysfunction. The findings highlight the importance of seasonal preventive measures to control TB infection in PLWHA, particularly those experiencing severe immunodeficiency.

Elevated levels of air pollutants are associated with impaired lung function due to oxidative stress, which may cause airway inflammation, inhibit the macrophage function, and increase susceptibility to *MTB* [58, 59]. And HIV impairs the host immune defense against *MTB* infection and impairs phagocytosis of *MTB* by macrophage [59], thus PLWHA are more likely to develop TB than general individuals. Furthermore, we found a significant detrimental impact of PM_10_ in PLWHA with severe immunodeficiency in present study. Therefore, it is reasonable to speculate that pollutants are more likely to induce an increased susceptibility and risk of TB morbility in individuals with severe immunodeficiency.

There are some limitations in our study. Firstly, the model was fitted using the average levels of weather, pollutants, and TB cases in nine cities in Guangxi province, which enhanced the overall stability of the model but impacted the estimation of the extreme effects. Secondly, given the ecological nature of this study, it is impossible to establish the relationship between the exposure and the effect at the individual level, and the ecological fallacy is inevitable. Therefore, this study mainly provides evidence for the associations between TB incidence, meteorological factors, and air pollutants in PLWHA, but it lacks evidence of causal inference.

## Conclusion

This study demonstrated the significant cumulative lag-response effects of temperature and precipitation on TB risk in PLWHA. Moreover, the hot, rainyt, and rainless effects are associated with a decreased TB risk in PLWHA. Additionally, the impact of meteorological factors on TB incidence is contingent upon the immunological status of PLWHA.

### Electronic supplementary material

Below is the link to the electronic supplementary material.

Supplementary Material 1

## Data Availability

The data generated and analyzed during the current study are available from the Chest Hospital of Guangxi Zhuang Autonomous Region, but restrictions apply to their availability. These data were used under license for current study and are not publicly available. But data are available from the corresponding author on reasonable request.

## Abbreviations

AIC: Akaike information criterion
CI: Confidence interval
DLNM: Distribution lag non-linear model
IQR: Interquartile range
PLWHA: People living with HIV/AIDS
TB: Tuberculosis
