# Supplementary material for: The effects of meteorological factors and air pollutants on the incidence of tuberculosis in people living with HIV/AIDS in subtropical Guangxi, China
Source: BMC Public Health. 2024 May 17;24:1333. doi: 10.1186/s12889-024-18475-0 (PMC11100081; doi:10.1186/s12889-024-18475-0)
Supplement: Supplementary file 1 — Supplementary Material 1 [file 12889_2024_18475_MOESM1_ESM.docx]

Table S1 The correlation between TB case and meteorological factors or air pollutants factors in Guangxi, 2014-2020.

| **Variables** | **TB case** | **CO** | **O_3_** | | **NO_2_** | | **PM_10_** | | **PM_2.5_** | | **Precipitation** | | **Sunshine duration** | **Temperature** | **Wind speed** | **Relative humidity** |
| --- | --- | --- | --- | --- | --- | --- | --- | --- | --- | --- | --- | --- | --- | --- | --- | --- |
| **TB case** | 1.000 | 0.040 | | 0.045 | | 0.043 | | 0.143** | | -0.020 | | 0.157** | 0.092 | 0.234** | -0.292** | 0.065 |
| **CO** | 0.040 | 1.000 | | -0.156** | | 0.713** | | 0.562** | | 0.647** | | -0.434** | -0.396** | -0.628** | -0.204** | -0.109* |
| **O_3_** | 0.045 | -0.156** | | 1.000 | | 0.135** | | 0.395** | | 0.268** | | -0.124* | 0.681** | 0.391** | -0.209** | -0.539** |
| **NO_2_** | 0.043 | 0.713** | | 0.135** | | 1.000 | | 0.791** | | 0.772** | | -0.503** | -0.120* | -0.536** | -0.359** | -0.349** |
| **PM_10_** | 0.143** | 0.562** | | 0.395** | | 0.791** | | 1.000 | | 0.925** | | -0.648** | 0.037 | -0.423** | -0.349** | -0.578** |
| **PM_2.5_** | -0.020 | 0.647** | | 0.268** | | 0.772** | | 0.925** | | 1.000 | | -0.725** | -0.136** | -0.592** | -0.252** | -0.531** |
| **Precipitation** | 0.157** | -0.434** | | -0.124* | | -0.503** | | -0.648** | | -0.725** | | 1.000 | 0.055 | 0.566** | -0.014 | 0.636** |
| **Sunshine duration** | 0.092 | -0.396** | | 0.681** | | -0.120* | | 0.037 | | -0.136** | | 0.055 | 1.000 | 0.661** | -0.191** | -0.398** |
| **Temperature** | 0.234** | -0.628** | | 0.391** | | -0.536** | | -0.423** | | -0.592** | | 0.566** | 0.661** | 1.000 | -0.150** | 0.197** |
| **Wind speed** | -0.292** | -0.204** | | -0.209** | | -0.359** | | -0.349** | | -0.252** | | -0.014 | -0.191** | -0.150** | 1.000 | -0.163** |
| **Relative humidity** | 0.065 | -0.109* | | -0.539** | | -0.349** | | -0.578** | | -0.531** | | 0.636** | -0.398** | 0.197** | -0.163** | 1.000 |

Note: *P*-values and correlation coefficient r were calculated using Spearman’s rank correlation, and *P* < 0.05 was considered to be statistically significant. TB = tuberculosis; PM_2.5_= particulate matter with an aerodynamic diameter less than 2.5 μm; PM_10_ = particles with an aerodynamic diameter less than 10 μm; SO_2_ = sulfur dioxide; O_3_=Ozone; CO=carbon monoxide; NO_2_=nitrogen dioxide; **P* < 0.05, ***P* < 0.01.

Table S2 The Q-AIC values of lag weeks for different variables.

| **Lag(weeks)** | **Mean temperature** | **Wind speed** | **precipitation** | **PM10** |
| --- | --- | --- | --- | --- |
| **1** | 1770.653 | 1679.962 | 1778.308 | 1697.364 |
| **2** | 1751.624 | 1670.339 | 1760.346 | 1687.424 |
| **3** | 1734.341 | **1664.158** | 1740.698 | 1687.424 |
| **4** | **1720.759** | 1667.602 | **1716.791** | 1667.430 |
| **5** |  |  |  | 1667.787 |
| **6** |  |  |  | 1666.516 |
| **7** |  |  |  | 1665.530 |
| **8** |  |  |  | 1660.224 |
| **9** |  |  |  | 1647.064 |
| **10** |  |  |  | 1643.758 |
| **11** |  |  |  | 1638.780 |
| **12** |  |  |  | **1621.806** |

Note: PM_10_ = particles with an aerodynamic diameter less than 10 μm; Q-AIC= quasi-Poisson Akaike information criteria.

Table S3 The sensitivity analysis results of different degrees of freedom for “time” for temperature is 22.5℃ in Guangxi, 2014-2020.

| **Lag(weeks)** | **df=2*7** | | | **df=3*7** | | | **df=4*7** | | |
| --- | --- | --- | --- | --- | --- | --- | --- | --- | --- |
|  | RR | LCI | UCI | RR | LCI | UCI | RR | LCI | UCI |
| 0 | 0.995 | 0.931 | 1.065 | 1.005 | 0.939 | 1.077 | 0.996 | 0.931 | 1.066 |
| 1 | 1.011 | 0.926 | 1.104 | 1.033 | 0.939 | 1.137 | 1.017 | 0.924 | 1.120 |
| 2 | 1.042 | 0.945 | 1.149 | 1.079 | 0.961 | 1.212 | 1.062 | 0.943 | 1.195 |
| 3 | 1.076 | 0.974 | 1.188 | 1.136 | 0.995 | 1.296 | 1.128 | 0.982 | 1.296 |
| 4 | **1.106** | **1.013** | **1.209** | **1.198** | **1.037** | **1.385** | **1.217** | **1.038** | **1.428** |

Note: df= degrees of freedom; RR= relative risk; LCI= lower confidence interval; UCI= upper confidence interval.

Table S4 The sensitivity analysis results of different degrees of freedom for “time” for wind speed is 2.05m/s in Guangxi, 2014-2020.

| **Lag(weeks)** | **df=4*7** | | | **df=5*7** | | | **df=6*7** | | |
| --- | --- | --- | --- | --- | --- | --- | --- | --- | --- |
|  | RR | LCI | UCI | RR | LCI | UCI | RR | LCI | UCI |
| 0 | 0.995 | 0.956 | 1.037 | 0.993 | 0.953 | 1.035 | 1.005 | 0.964 | 1.048 |
| 1 | 0.997 | 0.940 | 1.057 | 0.993 | 0.935 | 1.055 | 1.019 | 0.958 | 1.085 |
| 2 | 1.003 | 0.928 | 1.084 | 0.999 | 0.921 | 1.083 | 1.039 | 0.955 | 1.130 |
| 3 | 1.012 | 0.920 | 1.113 | 1.008 | 0.912 | 1.115 | 1.061 | 0.955 | 1.178 |

Note: df= degrees of freedom; RR= relative risk; LCI= lower confidence interval; UCI= upper confidence interval.

Table S5 The sensitivity analysis results of different degrees of freedom for “time” for precipitation is 6mm in Guangxi, 2014-2020.

| **Lag(weeks)** | **df=1*7** | | | **df=2*7** | | | **df=3*7** | | |
| --- | --- | --- | --- | --- | --- | --- | --- | --- | --- |
|  | RR | LCI | UCI | RR | LCI | UCI | RR | LCI | UCI |
| 0 | 1.048 | 0.934 | 1.176 | 1.023 | 0.910 | 1.151 | 1.035 | 0.923 | 1.161 |
| 1 | 1.149 | 0.979 | 1.349 | 1.132 | 0.952 | 1.347 | 1.156 | 0.972 | 1.375 |
| 2 | **1.277** | **1.055** | **1.546** | **1.299** | **1.040** | **1.621** | **1.335** | **1.064** | **1.675** |
| 3 | **1.365** | **1.102** | **1.691** | **1.440** | **1.107** | **1.874** | **1.488** | **1.130** | **1.959** |
| 4 | **1.361** | **1.087** | **1.704** | **1.481** | **1.108** | **1.980** | **1.534** | **1.124** | **2.093** |

Note: df= degrees of freedom; RR= relative risk; LCI= lower confidence interval; UCI= upper confidence interval.

Table S6 The sensitivity analysis results of different degrees of freedom for “time” for PM_10_ is 110μg/m^3^ in Guangxi, 2014-2020.

| **Lag(weeks)** | **df=3*7** | | | **df=4*7** | | | **df=5*7** | | |
| --- | --- | --- | --- | --- | --- | --- | --- | --- | --- |
|  | RR | LCI | UCI | RR | LCI | UCI | RR | LCI | UCI |
| 0 | 0.893 | 0.736 | 1.083 | 0.965 | 0.786 | 1.185 | 1.069 | 0.856 | 1.335 |
| 1 | 0.825 | 0.580 | 1.174 | 0.980 | 0.668 | 1.439 | 1.209 | 0.789 | 1.853 |
| 2 | 0.787 | 0.483 | 1.282 | 1.045 | 0.607 | 1.800 | 1.441 | 0.767 | 2.707 |
| 3 | 0.775 | 0.422 | 1.423 | 1.164 | 0.582 | 2.330 | 1.800 | 0.772 | 4.198 |
| 4 | 0.784 | 0.380 | 1.615 | 1.346 | 0.581 | 3.122 | 2.336 | 0.790 | 6.906 |
| 5 | 0.812 | 0.351 | 1.880 | 1.601 | 0.595 | 4.308 | 3.115 | 0.815 | 11.902 |
| 6 | 0.858 | 0.329 | 2.238 | 1.937 | 0.620 | 6.054 | 4.203 | 0.841 | 21.015 |
| 7 | 0.918 | 0.312 | 2.703 | 2.351 | 0.650 | 8.509 | 5.648 | 0.865 | 36.892 |
| 8 | 0.992 | 0.300 | 3.279 | 2.833 | 0.682 | 11.759 | 7.448 | 0.886 | 62.595 |
| 9 | 1.075 | 0.292 | 3.961 | 3.357 | 0.715 | 15.751 | 9.530 | 0.905 | 100.339 |
| 10 | 1.168 | 0.288 | 4.741 | 3.885 | 0.745 | 20.259 | 11.726 | 0.920 | 149.503 |
| 11 | 1.268 | 0.286 | 5.623 | 4.373 | 0.767 | 24.922 | 13.798 | 0.928 | 205.162 |
| 12 | 1.376 | 0.285 | 6.635 | 4.774 | 0.776 | 29.388 | 15.479 | 0.926 | 258.854 |

Note: df= degrees of freedom; RR= relative risk; LCI= lower confidence interval; UCI= upper confidence interval.
